# American Academy of Dental Science

**Published:** 1898-06

**Authors:** 

**Affiliations:** American Academy of Dental Science


### Reports of Society Meetings.

### AMERICAN ACADEMY OF DENTAL SCIENCE.

The regular monthly meeting of the American Academy of Dental Science was held at Young's Hotel, Wednesday evening, January 5, 1898, at six o'clock.

A paper, entitled "The Amalgam Question and Dentists with 'Yankee Wit,'" was read by Dr. C. H. Taft.

(For Dr. Taft's paper, see page 344.)

*Dr. Taft.*—Mr. President, before the subject is thrown open for discussion, I want to speak of a case which I had in hand last week.

A former patient of mine, who had removed to the western part of the State, where she has lived for the past five years, was visiting Boston, and came in for an appointment. She told me she had been having constant trouble with her throat for the past few years, and without being able to obtain any relief; that she had made arrangements, by the advice of her physician, to go to Southern California for a year. Her physician confessed to me that it was the worst case of sore throat he had ever seen. He had tried all kinds of remedies and treatments with no effect whatever, and she was unable to eat with any comfort because it was so painful for her to swallow. I made a superficial examination of the mouth and found the hard palate badly inflamed and the tonsils covered with ulcers. I remembered that I had several years ago put in three or four out of the eight or nine amalgam fillings that she had in her mouth, and I said to her after some little conversation, "I believe your trouble is greatly aggravated not only by the red rubber plate, but by all your amalgam fillings."

"That is just what my physician said," she replied. He was an allopathic physician, by the way, and had told her over a year ago that he had in mind sending her to her dentist to have the amalgam fillings taken out, but he thought that in any event a change of climate would have a very beneficial effect. I felt a great deal of interest in this case, and advised her not only to never put that red rubber plate in her mouth again, but to allow me to remove those amalgam fillings without expense to her. She agreed to this, and the work was completed day before yesterday. She came into my office yesterday morning and said to me, in a laughing way, "I certainly do feel better already, but whether it is due to imagination or not I cannot say. I haven't that 'blistered' feeling in my throat, and if it were not that arrangements have been so fully completed, I believe I should be almost tempted to give up going to California." I said to her, "If you are better, I am glad of it. I shall hope to hear, after you have reached California, not only that your throat is improving, but that you are fast gaining health, and I should advise you, when you go home again, to put that red rubber plate into the fire." She agreed to do it.

I shall be very much interested in watching this case, and will report to the Academy further in regard to it. If we find she gets better, we can attribute it to the imagination, to the change of residence, or to the removal of the amalgam fillings, whichever way we happen to feel about it.

*Dr. Allen.*—When Dr. Taft invited me to open this discussion of a somewhat unpopular subject, I suspect he did so because I represent some of the views of a minority of dentists and physicians who hold that the presence of mercury in amalgam fillings is, under certain circumstances, prejudicial to the health of the patient.

It is not my purpose to offer any new argument in support of the views so well presented by our essayist in the paper just read, as well as in those presented by him on previous occasions. His plea for a calm consideration of the subject, based on clinical and experimental investigation, is the point which I desire to emphasize, and which ought now to guide us in our discussion. Let us approach the question as students, seeking whatever truth lies hidden therein, for in doing this we shall only follow the rational methods of the lover of science, and thus avoid useless controversy.

I do not profess to have wholly discarded amalgam from my practice. I occasionally find it useful in spite of its many defects as a filling-material, and shall doubtless use it more or less until a satisfactory substitute is found.

The susceptibility to the effects of mercury, as well as to other drugs, varies greatly with different individuals, and perhaps with the same individual under varying circumstances. It may be the exception for one of our patients to be at all affected by the mercury contained in amalgam fillings; but the fact appears to be well established of persons having been even seriously affected by this substance, which has been so indiscriminately used in the mouths of our patients by many members of the profession.

Our use of drugs being rather limited on the whole, and largely confined to local applications, we cannot claim to be expert observers of their more obscure physiological effects. On the other hand, when the physician, whose business it is to know just what a drug is capable of accomplishing in the human organism, has reason to believe that his patient is susceptible to small quantities of mercury, the dentist, unless he possesses superior knowledge of that particular case, should hesitate before assuming the opposite opinion.

We have had the experience in our dental meetings of hearing this subject treated with ridicule, and with expressions of personal prejudice, *pro* and *con*, much to the regret of those who appreciate the efforts and motives of the intelligent truth-seeker. It is to be hoped, therefore, that we will not pass this subject without a show of interest in its truly scientific aspect.

*Dr. Bradley.*—Whether we agree with the essayist or not, I think we shall all be willing to give him credit for a paper written with the purpose of presenting this subject fairly and judiciously. I was surprised to learn from his statement that there were dentists who would pretend they were filling the teeth with one material while they were using another. No conscientious dentist would do such a thing. Whether we agree with the writer's theory or not, we should let the patient know, if he asks for the information, what we are doing for him, and without any ambiguity in terms. If a patient desires that his tooth shall not be filled with amalgam, we should do the best we can with some other material. We certainly should not deceive him, for it is not professional.

In the course of a conversation with a prominent physician of the old school, he asked me about rubber plates and whether I ever noticed any ill effects from them. He gave it as his opinion that no person should ever wear a rubber plate; that they were exceedingly injurious to the membranes of the mouth, and he drew for me a diagram of one made from compressed paper. He said that they now make almost everything of compressed paper, and it would be lighter and would not poison the mouth. He was very sure that the rubber with mercury in it was injurious to the throat. Dr. Taft claims that the objection to mercury is not confined to the homœopathic school, and I merely speak of this as one instance corroborating his claim. I use amalgam fillings, and believe they are a great help in saving the teeth, and yet at the same time believe in the homœopathic system of treatment. I would like to know more about this. I believe that amalgam fillings are valuable in saving the teeth, and yet if my patients requested me not to use amalgam, I should comply with their desires and substitute something else. I would try to be honest with them, at all events. As Dr. Taft quoted very pertinently, I would be "true to myself," and in that way would do them no wrong.

*Dr. Barker.*—I have been wondering whether it was because of the dislike the members have of revising the stenographer's notes that they refrain from saying anything on this subject. Manifestly, the only way we can tell whether strawberries are going to poison us or not is to eat them. If they do, we shall be obliged to give up eating them. Manifestly, also, the only way we can tell whether amalgam fillings are injurious to a person or not is to put them in and note results. If they do prove injurious, really and actually, or if some physician thinks they are, then we can take them out and do the best we can with other materials. But I submit that until it can be proved that strawberries are injurious to the human race, as a whole, we shall continue to cultivate and eat them, and I submit likewise, Mr. President, that until it can be proved that amalgam fillings generally, or even in a small minority of cases, are injurious, the dentist who has a general practice to conduct and who has to take into consideration a variety of things, and among others the ability of the patient to pay fees, will be compelled to use dental alloys.

I have not the slightest disposition to ridicule this subject. I want to hear all the objections that can be brought up against amalgam by those who are making a study of it; it is certainly a subject the Academy ought to consider.

Is there any way by which the mercury we combine with an alloy and insert in a tooth can do injury, except as it shall become volatilized and taken into the system, and cause a loss of weight in the filling. Has any one inserted in a tooth an amalgam filling the weight of which he knew absolutely, and, after a time, taking into account the loss which would result from attrition, has he carefully removed the filling and ascertained whether it had lost weight, and if it had, has he ascertained whether a disproportionally large amount of mercury is lacking or not?

I remember reading somewhere the report of a committee appointed to investigate the possibilities of injurious effects from red rubber plates, and their report, after having made a thorough and exhaustive investigation, was to the effect that there was no evidence whatsoever, that they were able to obtain, that the mercury that had been present in the rubber plate had been lost after a certain length of time. The mercury did not get away from the plate and get into the system, and if it did not, how could it do harm? Does the mere presence of the mercury, either alone or combined with the alloys, produce unfavorable results? If it does, then, of course, we ought to determine in the case of each individual patient who presents himself to us for our services whether the risk to that patient is sufficiently great to justify us in denying him the use of amalgam, and in substituting therefor gutta-percha or zinc phosphate, or in being charitable and putting in gold. The essayist admits that there is no general over-susceptibility to influence of mercury, or to amalgam fillings, and it occurred to me that, as we save thousands and tens of thousands of teeth every year by their use, we had better continue their use until we find a case where there is pretty good evidence that they are poisonous to a certain patient, then let us remove them and put in whatever the patient demands or whatever they can pay for.

It is an old subject, it has been threshed over and over, and yet I presume there are some grains of wheat yet in it, and I am glad to see the Academy is willing to try to find them. Let us meet the question in a fair and candid spirit, and I have no doubt we shall find some profit in the discussion of it.

*Dr. Andrews.*—I wish in the first place to congratulate the essayist on the very admirable way in which he has presented the subject to us.

I shall speak for a moment on one phase of this question, and that is, the power of mercury to penetrate minute canals. It is a fact, gentlemen, that globules of mercury are forced into the dental canals, and in the case of pulpless teeth forced through the canals to an incredible extent. It is perfectly surprising how mercury will travel into minute openings. Some of you have bought your mercury in the little wooden bottles as put up by some of the supply houses. If you split these bottles open, you will find mercury running all through the grain of the wood. I remember having shown the Academy, in one of my demonstrations on tooth-structure, a section of a tooth that had been filled with amalgam, a section having the canals of the dentine completely filled with the minute globules of mercury; now, when we reflect that those canals are only about one-ten-thousandth of an inch in diameter, we can see that mercury forces itself into very minute openings. I am making a series of investigations on freshly extracted teeth, that had live pulps when they were extracted, with an idea of finding whether these globules will penetrate into the canals when the fibrils are still present. I will give the results of these investigations at a later meeting. I have here one of the wooden bottles which has held mercury. I shall pass it around with a magnifying glass, and you can see for yourself the globules of mercury in the grain of the wood. It looks as if you had taken a lead-pencil and drawn a very fine line in the interior of the wood. When I discovered this fact, I notified some of the manufacturers that, by placing the mercury in the wood just as it was turned, they were really wasting some of their mercury, and we were not getting the benefit of all we paid for, because the mercury was forcing itself into the substance of the wood. There has since been an effort on the part of the bottlers to guard against this by varnishing the inside of the wood with some thin varnish and letting it dry. It was thought that by doing this it would fill up the pores of the wood and make a surface that would not take mercury. I show you to-night, however, some bottles that have been varnished, and the mercury still runs down into the fibres of the wood. I have mounted a number of sections taken from different pieces of these wooden bottles; in some of which the openings are quite minute, but they are completely filled with mercury. It has been my practice for several years when filling teeth with amalgam to line my cavity, and I think it is the best practice. It not only gives you a clean tooth, but prevents the possibility of the mercury going into the tooth-structure. I use amalgam where I think its use is indicated. I tell my patients before using it that there are objections to its use by some physicians. I tell them that I do not believe such fillings can do harm. At the last meeting of the dental section of the American Medical Association, held in Philadelphia, the subject of amalgam fillings was brought up by Dr. Bonwill. He wanted very much to have a committee from the dental section examine the mouths of some of his patients. It was voted to have a committee call at Dr. Bonwill's office the next morning and make the examination. At the appointed time the committee met at Dr. Bonwill's office, and found that he had collected about forty persons, the majority of whose teeth were found filled with amalgam, and Dr. Bonwill wanted to show that amalgam, when properly used, would not produce ill effects. I do not think there were ever seen a healthier set of mouths, and this was the unanimous voice of that committee.

I would urge that before you put in amalgam fillings you would line each cavity with some kind of cement. In the cases of large fillings I make a practice of filling the cavity partly with oxyphosphate, packing the amalgam into this. I think it is on record that gold is a dangerous article to use in the mouth in some cases, and I remember reading a paper describing the bad effects which the writer had noticed caused by filling teeth with oxyphosphates and oxychlorides. It may be that there are some such cases, but I doubt very much if amalgam, properly used, will do any harm in the large majority of cases.

*Dr. Banfield.*—May I ask Dr. Taft, if he entirely discards the use of amalgam because of these few cases in which he has noticed bad effects resulting from its use?

*Dr. Taft.*—I have practically discarded it. I do not use on an average an ounce in a year.

*Dr. Banfield.*—If that is so, Dr. Taft goes a step further than physicians in the use of drugs. Not long since being lame and sore from the effect of a cold, various remedies were tried without giving relief; finally rhus tox. cerate was applied externally.

This remedy had been used by others through the advice of a physician for rheumatic troubles and had produced no disagreeable effects. The following day wherever the application had been made an eruption appeared, which caused much suffering and confined me to my bed for several days. I afterwards made inquiries of Otis Clapp & Co., manufacturers of this drug, and learned from them that occasionally similar disturbances arise, but that as the cases are so few physicians do not consider it necessary, in prescribing it, to especially caution patients in its use.

Now, here is a drug that does irritate in some cases, and is a poison to some persons, but because of these few, isolated cases physicians do not discontinue its use, so I do not see why we should discard amalgam because it creates a disturbance in a small percentage of cases.

*Dr. Smith.*—This is the old case of amalgam on trial. Certain medical practitioners allege that amalgam fillings are poisonous, injurious, and should not be used. They have settled that matter for themselves, and from their stand-point it should no longer be discussed. I understand a bill has been, or will be, presented to this Legislature, prohibiting dentists from using amalgam as a filling. No dentist, if he can be convinced that amalgam is injurious, would wish to use it; in fact, from a pecuniary stand-point, it would be beneficial to him if he could not use it, because it means in cases where gold is contraindicated that he has the opportunity to fill and refill and fill again. Therefore, from that stand-point, from the sordid stand-point of gain, he should support that bill. But, on the other hand, gentlemen, see what hardship it works upon the poorer class of people who cannot afford gold and are obliged to undergo the expense of replenishing cements and gutta-percha, and none here, I think, will deny that the cements and gutta-perchas do need replenishing, and oftentimes at the expense of the tooth-material.

Now, to prove this claim that amalgam is injurious these gentlemen cite cases similar to those which the essayist has cited in his paper,—cases where they have treated certain troubles with, as I presume, the proper remedies, and, finding no headway, have had amalgam fillings removed, with the pleasure of seeing the patient go on to a speedy cure. Now, what is the offset to those cases? In my practice I have had two, of which I will speak, and possibly, if we are observing, we may find still more evidence in the same line.

A certain patient was in my hands for the care of her teeth, and also in the hands of a prominent homœopathic physician for some other trouble; knowing the fact that she was being treated by a homœopathic physician, I never thought for a moment of using anything in her mouth but cements and gutta-percha. That patient continued under treatment for two years. If that patient had had amalgam fillings, they would have been ordered out as being the cause of her trouble, and had she recovered in that time, the conclusions would apparently have justified this belief. Circumstances called her to another city, where she was treated by an equally prominent homœopathic physician, and one who supported the high potencies with as much fervor as her physician here. She also came into the hands of another dentist, who, probably, knowing nothing about the objections of homœopathic physicians to amalgam, removed the cements and gutta-percha and replaced them with amalgam, so that when she came under my notice again I was surprised to find these molars well filled with amalgam, and the patient was perfectly well. Now, if the argument is good on one side, that amalgam fillings prevented the cure, then that same argument holds good on the other side,—that putting in amalgam fillings cured her. To my mind, if one is good logic, the other is equally good logic, and I do not see how you can escape it. I have had two such cases.

I think that this charge against amalgam is still unproved; but I do admit that there are idiosyncrasies on the part of some people, and that they may be susceptible to amalgam, and that it will affect them in peculiar ways. We also know that bicarbonate of soda will produce a severe inflammation of the mucous membrane in some cases, yet we would not go before the Legislature with a bill prohibiting the use of bicarbonate of soda as a mouth-wash.

From these high potentists comes the assertion that a large number of suicides are caused by the presence of gold in the teeth.

I am merely stating facts, gentlemen,—and if to state facts is to ridicule, then let it be so. Aurum is a specific for certain mental troubles, and it is believed by certain physicians that so much gold in the mouth tends to produce these troubles. Then we are told that by removing salivary calculi from the teeth they drop out. Again, some physicians say that a fistulous opening should not be cured. These physicians will not allow the dentists to use drugs for curing them, because they claim that it is a condition that nature has established to relieve impurities, and, therefore, the drainage should be allowed to go on. I have heard physicians make this statement. And now, Dr. Andrews says that charges have been brought against oxychloride and oxyphosphate. Well, if all these alleged charges can be sustained,—that amalgam is injurious; that gold is injurious; that the removing of tartar must not be allowed; that in our treatment of abscess we must not attempt to close up a fistulous tract, then "Othello's occupation is gone."

Now, I use amalgam in my practice, discriminately, as I use all other fillings and drugs, and I believe, with the essayist, that no man would be justified in deceiving a patient as to the material he was using because he himself did not believe that material would injure the patient.

To refer to another case of mine in which amalgam fillings figured as the bone of contention: the patient's teeth were badly decayed and I had filled them with cements and gutta-percha until I was thoroughly discouraged, and I suggested to this patient that she allow me to put in amalgam, knowing that her physician was an allopathic physician who would probably not object to the use of amalgam, and she did allow me to do so. She is a woman who abuses herself in following fashionable society, and in the midst of a society season she suffers more or less from stomach trouble and always did, and I have felt that her distress was due to the abuse which she gave herself rather than to any lack of physician's skill to cure her. Not getting relief which she looked for, she broke loose from the allopathic physician and went to a prominent homœopathic physician for medical advice. The first thing he asked was if she had any amalgam fillings in her mouth, and on receiving her reply in the affirmative, he told her she must have them removed at once. She returned to me, and wanted them removed at once, believing them to be the cause of her not gaining her health. I told her that I did not believe the amalgam fillings had any effect whatever upon her health, and that their removal would be simply a matter of more expense and more trouble to her. I removed those fillings and put in cements and gutta-percha again, and she followed the treatment of the homœopathic physician, but without obtaining the desired relief. Finally, she went back to her allopathic physician, and after a time began to improve. Now, if she had improved in health while under the treatment of that homœopathic physician, she would have alleged that the amalgam fillings were the cause of her keeping in poor health, but in this case, even after the removal of the amalgam fillings, she continued in ill-health.

Speaking of inflamed spots and ulcers, I have seen them caused by ill-fitting rubber plates, and you will see that same inflammation under ill-fitting gold plates.

*Dr. Werner.*—I feel that every one of us would like to speak on this subject, and it would take a long time to express all our notions and partial knowledge on the subject. Were I to ask the members here if they generally believed that there can be free mercury in an amalgam filling, most would reply, "No." I was of that opinion, but lately I have been convinced that there is free mercury in some amalgam fillings. I have pieces of gold here that touched an amalgam filling and will pass them around. If you believe in homœopathy, you will naturally believe that an amalgam filling that had free mercury would be injurious. I do not wish to ridicule homœopathic methods and principles, but if I were sick, I would not expect much help from a thousandth part of a drop of medicine. I remember an excellent article by Dr. Holmes in the *North American Review*, on the "Fallacies of Homœopathy." It is too early for all truth to be known. Once we did not believe in bacteriology, though I believe some one in Germany, back in Hahnneman's time, did believe in it.

On May 8, 1897, I was filling a right inferior third molar, mesial and crown cavity with gold. In the right inferior second molar, distal and crown, was a large copper amalgam filling which was put in perhaps fifteen years ago, it looked a good deal worn. I had adjusted the rubber dam and matrix, and my assistant was passing the gold into the cavity, and in doing so would pass the gold cylinders over the amalgam filling in the second molar, accidentally touching the amalgam with the gold. Soon I found that my gold filling rocked, and there was noticeable discoloration of the gold after being condensed. I turned to my assistant and asked, "What have you on your foil-carriers?" She replied, "Nothing." I told her it seemed as if she had either iodine or mercury on the foil-carrier, as the gold was loose and discolored, and that we would have to start over again. The foil-carrier was cleaned thoroughly, and I proceeded the second time, but soon found that my filling was again rocking; the gold cylinders did not cohere. I took it all out again, and have it here in this bottle and would like you to see it. Afterwards, with a clean pair of foil-carriers, I took cylinders of gold and passed them over the amalgam filling in the second molar, and the gold became literally covered with mercury, as you see it in the bottle. Now, that patient is not very sick, and has never, according to my belief, been injured by that amalgam filling and its free mercury. She is more or less dyspeptic, and I have no doubt a homœopathic physician would consider it an excellent case of direct poisoning from amalgam filling.

I believe this presentation of the subject as given to us by the essayist is the most scientific, most logical, of any we have yet heard, —providing you believe in homœopathy.

*Dr. Allen.*—May I interrupt the gentleman for a moment? I suppose he intends his remarks to refer equally to the allopathic physician who believes that amalgam fillings are injurious, and there are a great many who do.

*Dr. Werner.*—I doubt very much the scientific basis on which these objections stand at the present day, whether they come from the allopathic or the homœopathic physician. I do not doubt but that they think they find the symptoms the essayist describes, but are they real or imaginary, and are they due to the presence of mercury in the amalgam fillings? That is the question.

As referred to before this evening, there are many people who cannot afford to have gold, nor can they have their teeth patched up with cement or gutta-percha every six or ten months, and hence they are better served with amalgam fillings. In my practice I use perhaps as little amalgam as any man here, for I believe in gold in distinction from amalgam fillings, and my patients think the same. I might perhaps attack the essayist stronger yet, although I think he sincerely believes what he has told us.

*Dr. Taft.*—Do not be afraid, doctor; I want to hear whatever you think about this subject.

*Dr. Piper.*—One point in Dr. Barker's remarks recalled to my mind something that happened a short time ago. A young lady was suffering from some trouble, and the gentleman to whom she was engaged advised her to go to his physician, a prominent practitioner not far from Copley Square. One of the first things he asked her was, "Have you any amalgam fillings in your mouth?" She replied that she thought she had, and he told her to go to Dr. —— and have them removed. She asked why she could not go to her own dentist. "Because," he said, "I am afraid if you should go back to your own dentist, he would tell you that he did not think it was necessary to remove these fillings, and advise you against it." She became indignant, and told him that she had confidence in the ability of her own dentist to take care of her teeth, and declined to go to the other man. She came to me and told me the facts and said, "I want you to go down and talk with him, and find out, if you can, why it is necessary to have those fillings removed." So I made an appointment, and early one morning went down to his office. I talked nearly an hour and a half to this man on the subject, and about all that I could get from him that I could understand was that the trouble was caused by the "dynamic effect." It did not seem to be due to absorption of the mercury into the system. We all know what dynamics are, but what the meaning of the dynamic effect of amalgam fillings in the mouth is I do not know, and I could not get any satisfactory explanation from him. I would like to ask Dr. Taft if he can make it clear to us.

*Dr. Eddy.*—The only point I would speak upon is in regard to the value of medical opinions. We have two cases in the Rhode Island courts involving this question. In a case of suspected diphtheria the physicians for the patient claimed that there was no diphtheria, while the representative of the State Board of Health said there was diphtheria. Both judgments were given on evidence of the examination of cultures. The house was placarded, and, as a consequence, suit was brought against the board of health for restricting the liberties of the occupants. So I do not know whether we are right or wrong in following physicians' advice in certain cases.

*Dr. Briggs.*—I am sorry I did not arrive in time to hear the paper. I do not know whether the essayist made this a homœopathic issue or not. If he did, I do not care to discuss it. Or, if he laid down the law that the mercury of amalgam fillings, as a rule, was injurious, I do not think that is a discussable point with the material we have at our disposal. On the other hand, it seems to me that if the question he has brought before us is, "Can amalgam fillings injure a person?" I think that is open to discussion, and I am not prepared to say that they do not. Any one who has had any experience with drugs, foods, or medicines of any sort knows the possibility of meeting with idiosyncrasies which may exist at certain times in some individuals, and at all times in other individuals. We know that there are persons to whom gold would be irritant poison; we know that all the metals are severe poisons in excess of the therapeutic dose, and that an individual could not be found here and there who would be affected by the different metals that we use I would not attempt to say. But then comes the question, "Why is it that mercury is selected as a scape-goat?" It seems to me from the cases that I have observed, where I have admitted to myself the possibility that amalgam fillings were injuring a person, that the irritation, the possible danger of poisoning, came quite as much from the metals contained in these amalgam fillings as from the mercury. This has led me to feel, in my practice, that a properly made, properly put in amalgam filling was not dangerous; whereas, I have taken out amalgam fillings that looked to me as though they might be dangerous. When you see a filling that is constantly and steadily losing substance, you know that the ingredients are going into the patient, and if it happens to be one of those few patients who are peculiarly susceptible, it may be doing harm. I have taken out such fillings.

I think if this thing is going to be discussed, we must discuss it from all its points and not confine ourselves to the mercury. I have removed most of the copper amalgam fillings that I have put in, but in those cases where I used the high grade alloys, properly mixed, and properly put in, I have yet to see any case where there was any sign of trouble or any cause for their removal.

I remember the case of one patient who had a sore throat and consulted a homœopathic physician, who told her the usual story that nothing could be done until the amalgam fillings were removed. I told her what I thought, but at the same time said I would be very glad to take them out if she wanted to undergo the necessary trouble and expense. I told her that in all the amalgam fillings in her mouth I could see no signs of danger from any except possibly one copper amalgam filling which had lost half its substance, and if she were susceptible to the poison of mercury or to the poison of copper, that was undoubtedly the one which was causing the mischief, and I would be very glad to remove that one and see what the result would be. I did that, and she reported after a while that her throat was very much improved, and later she came to me again and said that she wanted the rest of them removed. I removed them and put in gold fillings, but there was never any further improvement in her case. We must admit, of course, as scientists, the existence of these idiosyncrasies, but if you are going to begin to discard materials because of idiosyncrasies, you must discard your gold and also the phosphates. Fill your teeth with intelligence and judgment, and watch your cases, and if there is trouble, look to see what the trouble is, and if you have reason to think it is caused by amalgam fillings, remove them, but I do not think it is necessary to go into an absolute condemnation of amalgam. I have not, and I shall not.

*Dr. Potter.*—We learn from experience that physicians know very little about the teeth and the affections thereof, and hence a prevailing scepticism as to their opinions upon dental matters. When they show us that they have studied the teeth and know them well, I think we will be able to place more confidence in what they have to say about amalgam fillings and rubber plates. I agree with what Dr. Briggs has said as to the possibilities of harmful results from the use of amalgam.

*Dr. Barker.*—I want to get some information if I can. We want to discuss this question with a view to forming opinions. Will the essayist or will any one tell me on just what ground the belief is based that amalgam fillings are injurious; is it upon the ground that free mercury passes from the filling into the system? As supporting that view is the illustration of Dr. Werner, but with all deference to his statement, it seems to me there must have been some mistake, and there must have been some other cause for his filling "rocking" which he did not understand. Suppose I have an amalgam filling that has been in the mouth for ten years and presents a good, clean, nicely polished surface, how many dentists here present, how many members of this Academy, would hesitate to put gold right into that amalgam filling supposing it to be dry? I would not. My confidence might be misplaced, but I should not have any expectation that free mercury would pass from that amalgam filling into the gold. If my filling "rocked," I would not believe it was because of having absorbed free mercury, but from some other cause. I believe an amalgam filling becomes crystallized, and that no free mercury passes out of that filling either into the stomach of the patient or any where else.

*Dr. Taft.*—I will answer Dr. Barker's question by asking him another. I do not pretend to *know* or to be able to explain to the satisfaction of every gentleman in this room just what the dynamic influence or action of a drug is. To my mind it is a force of some kind,—an invisible, spirit-like force. Now what is it that constitutes the difference between a live man and a dead one? Is it not the presence or absence of what we call the *vital force?* When a physician is treating a sick man, he finds a condition where the vital force is at a low point, and he attempts to restore it by the use of drugs, or, I should say, by means of their *dynamic*

force. Does he expect that, unless they possess some inherent force, the drugs are going to have the slightest effect upon the material organs or tissues of the body? If that is the case why cannot he successfully treat or resuscitate a dead man with the proper medicines?

In regard to the dynamic influence, therefore, I cannot tell you beyond this what it is, but I would like some one to explain to me why it happens that some persons cannot pass near a bed of poison ivy without becoming badly poisoned. Is there any "free" ivy which gets into the system? If not, is there anything in the force or energy emanating from that poisonous plant that we can see, measure, feel, taste, weigh, or smell?

It has been asked, Is there any free mercury going off from an amalgam filling into a person's system? I am not prepared to say that there is, but I would like to ask any gentleman here who has had occasion to take off a gold band surrounding a tooth that had been filled with amalgam, if, when he removed the cap, he examined that gold band? I have had occasion to do it, and have found the gold band perfectly rotten clear through. I have found that gold caps where they come in close contact with an amalgam filling will present a worm-eaten appearance. But who shall say whether this is due to free mercury, the vapor of mercury, or to some other cause? There are lots of things of which we cannot explain the why or wherefore.

We must reason inductively. We observe certain facts; and from continual observations we come to establish general principles; after these principles are established, we bring them down and apply them to particular cases. Now, in the case of the gentleman who cannot be in a room where there is an uncorked bottle of the bichloride of mercury without developing a severe headache, what is it that causes that headache? Is it free mercury that is going into his system, or is it the *dynamic effect* of the mercury upon that person's vital force? The gentleman does not attribute it to his imagination, because when the bottle is removed the headache, sooner or later, disappears.

How do we explain the cessation of chronic headache after the removal of amalgam fillings? I have seen such headaches entirely cured after I had faithfully taken out every particle of amalgam. Perhaps some member can offer an explanation. I think the Academy has approached and discussed this subject in a very fair-minded way, and with a desire to get at the truth, and I feel that the evening has been a profitable one to us all.

*Dr. Eames.*—Will Dr. Taft please inform the Academy in regard to the proportion of individuals who are thus affected by mercury.

*Dr. Taft.*—I do not find that it is a large number. I meant to convey that idea in my paper. I simply wanted to say that I believe a susceptibility to mercury may be established at any time. Mercurial or other abnormal symptoms may appear at any time in a person who has never exhibited them before. That is true of ivy, arsenic, or other harmful influences. We may not be susceptible to-day to the toxic effect of these poisons, but it may happen that to-morrow or next week we will be.

In this connection, I remember a point that I wanted to speak of, a remark made by a chemist who was in my office not long ago. In mixing an amalgam filling, I allowed some of the mercury, in squeezing the mixture, to drop on the carpet, and he asked, "Do you not think that is rather a risky thing to do?" Asking him for the reason why, he replied, "When mercury becomes very finely divided into such a number of minute particles as have fallen on the floor it takes but a moment for the vapor to fill a room like this at the proper temperature, and this vapor is poisonous. Where these globules are gathered together, making one large globule, the volume of vapor would of course be greatly diminished."

*Dr. Eames.*—I would like to ask Dr. Taft what he believes to be the *modus operandi* of the poisonous action of mercury? Is it a dynamic action?

*Dr. Taft.*—There is an influence there, whether it is the dynamic influence or not. I do not see why it should not be.

*Dr. Eames.*—As I understand it, Dr. Taft does not claim that he can explain whether a minute quantity of mercury is absorbed by the system and produces this effect, or whether it is a dynamic action.

Physicians of the homœopathic school are divided on this point; some of them do not believe at all in this dynamic force. I have been endeavoring to get an acquaintance of mine, a homœopathic physician, to come here this evening. He believes sincerely in the theory of the dynamic action of drugs,—that it is the mere presence of mercury which produces untoward effects, and not free mercury getting into the system, but he did not think it wise to come and state his opinions. He said, "At present we are not prepared to show how this is, but we believe that later we can show some scientific reasons why this is so, and give the proportion of cases thus acted upon in the observation of a certain number."

As I understand it, the proportion is acknowledged to be small, and it does seem unreasonable to abandon the use of amalgam altogether just because of the possibility of danger in rare cases.

The cases of strawberry-poisoning, of which Dr. Taft speaks, are properly termed idiosyncrasies. I know a man who cannot eat a dry cracker without having an area of about an inch square break out into perspiration on his left cheek. A young lady cannot eat eggs without vomiting. A school-teacher cannot take a teaspoonful of honey without his neck swelling to such an extent as to cause great difficulty of breathing, and causing the most distressing symptoms. I have heard of several persons in whom opium produces catharsis instead of constipation. Sometimes nitrous oxide cannot be inhaled, while ether may be taken with impunity. I know of an instance in which ipecac, being compounded in the third story of a house, affected a person on the first story. I know of a lady in this city who sold a horse because she could not ride behind him without being affected with asthma; and a patient of mine has the same trouble with regard to dogs,—he cannot have one without being affected with asthma. In all these cases there is a peculiar condition of the system by which food, drink, or medicines act in a peculiar manner, and those proportionately few cases of idiosyncrasies should not affect our general use of a material or drug which we know to be beneficial in the vast majority of cases.

A word in reference to the action of mercury upon gold. I could not help thinking of the beautiful work which we see in combination fillings as shown us by Dr. Clapp. In some cases the cavity is nearly filled with amalgam, and then gold is packed in to finish the filling, and yet it does not injure it. I agree with Dr. Barker that I should not hesitate to put gold in combination with amalgam where I thought the case would be better served by so doing.

*Dr. Taft.*—I would like to ask if any one of the members have noticed little holes in gold bands, whether upon a tooth which is filled with amalgam, or in gold caps which are in close proximity to an amalgam filling?

*Dr. Briggs.*—I was going to speak on that very point, because I took one off last week that I placed on ten years ago, and in that case the tooth was filled with amalgam. The tooth had decayed still more, and I took off the band and placed on a gold crown, but there was no trouble with the band,—it was just as hard as the day it was put on. I might say that I have repaired little places in gold caps with alloy, and in such cases, and, in fact, in all cases, I always like to use a high grade alloy. I do not see anything remarkable in the cases which Dr. Taft has cited where persons have been cured after the removal of amalgam fillings, but his reasoning is a little specious when he attempts to deduce from them that we should not use amalgam. We know that drugs are volatile; many of them are exceedingly so, and some of them are liable to affect certain persons by their mere presence. This is a fact with the ivy-poison. I know a physician who cannot handle an uncorked bottle of rhus tox. without getting all the effects of ivy-poisoning, but that simply shows his idiosyncrasy. He uses this drug for his patients whenever it is indicated.

How many of us see cases of ulcerated sore throats? I do not recall more than a very few in twenty years' practice, and in the worst one I ever saw there was not an amalgam filling in the mouth, but there were some abominable gold fillings. I took them out and replaced them with proper gold fillings, and the ulcerations disappeared. It would have been a characteristic mercury mouth if there had been amalgam in the teeth. Another case was that of a very sensitive patient, for whom I had placed in a bridge. She had not permitted me to grind the crowns down as much as I wished to, and she afterwards came to me with a very sore throat. The removal of the bridge and placing her teeth in good order cured the sore throat. On the other hand, I remember the case of a lady who was sent to me to have her amalgam fillings removed, because she constantly suffered from headaches and her physician could not effect a cure. I took out all the fillings and replaced with other materials, and she still suffers with headaches.

*Dr. Taft.*—I would like to ask Dr. Briggs in how large a percentage of patients of mine who have come to him to have amalgam fillings removed has he noticed this dissolving away of copper amalgam fillings that I had inserted?

*Dr. Briggs.*—I did not mean to intimate that there were a great number of them, but in the few cases that I have seen, I thought it better to remove them because they had shown this wearing away. It occurred to me that if they were dissolving, the patient ought not to be swallowing copper.

*Dr. Taft.*—How extensive was the dissolution in those that you did remove?

*Dr. Briggs.*—I should say all the way from an eighth to a third.

*Dr. Taft.*—I want to say, Mr. President, that I see a considerable number of my former patients, and if it were not for my convictions with regard to mercury, I should say that I can see not the slightest reason for discarding the use of copper amalgam. The fillings that I put in ten years ago I find to-day to be in absolutely perfect condition, and, furthermore, preserving the teeth perfectly, and I can recall to mind but two cases where I have seen any marked dissolution of copper amalgam fillings that I have inserted. The reason I asked about this dissolution was because I think there are some of our members who were present at a meeting of the Harvard Odontological Society a few years ago, where Dr. Briggs spoke of my having left behind me, upon my removal to Chicago, a great many cases where I had inserted copper amalgam fillings. He is on record as stating (see February, 1894, issue of the International Dental Journal, p. 119) "that it had been his privilege to see some of those fillings, and that he could testify that I seemed to have done some great work in preserving teeth by the methods I had followed."

*Dr. Briggs.*—I took one out only a month ago that had been gradually disappearing ever since it first came to my notice.

*Dr. Taft.*—I have never been obliged to remove a copper amalgam filling that I have put in for the reason that it showed this dissolution which so many complain of; but I am continually taking out alloys of tin and silver for the reason that the teeth are so manifestly going to pieces under these fillings. In most mouths, I am free to say, the amalgam fillings are a disgrace to whoever put them in. They show a shrinkage or contraction of the metal about the margin, and on removing the filling you will find the cavity is generally twice as large as it was when the filling was placed in; but I have yet to see a copper amalgam filling that I myself have placed in where there has been any such shrinkage or contraction. They are all as hard as flint. Copper amalgams seem to throw out something like tendrils into the tubuli of the dentine, and in order to remove one you have to cut and scrape until you get down to the very thinnest layer. It adheres like cement to the walls of the cavity,—even more firmly.

*Dr. Andrews.*—Is it not true that there is more mercury in a copper amalgam than in the other alloys?

*Dr. Taft.*—Not if you squeeze the mixture very dry to get out all the mercury possible. It takes them about a day to set, when they are placed in as dry as you can get them. If the mixture is inserted in a very plastic condition it will set in about fifteen minutes, and I hope if you decide to use copper amalgam to any extent that you will be sure to remove all the mercury that you can, and you will find that they will then be very much more durable.

*Dr. Briggs.*—If the essayist would start a crusade against those men who put in amalgam fillings so carelessly, make improper preparations, and do slovenly work, why, I am hand and glove with him. That is what has cast the odium on amalgam fillings. Many dentists seem to think that when placing in amalgam anything will do in the way of preparing the cavity. I do not see any reason why you should not prepare the tooth as well as you would for gold. I do not say that I always do that myself, but I am doing better every day.

*Dr. Maxfield.*—There is one thing I would refer to. I dislike very much to have the impression go out from this society, as it may when the proceedings are published, that amalgam fillings are used simply because the patient cannot afford to pay for gold. It seems to me we should use amalgam, gold, gutta-percha, or any other filling, because they are the best things to use in the particular case which we have at hand. I believe, in the majority of cases where we use it, amalgam is better for the patient than a gold filling. I do not believe in subjecting the patient to four or five hours in the chair when I can do practically as much good by inserting an amalgam filling in less than an hour.

In the matter of free mercury, spoken of by Dr. Werner, he has not given us any scientific basis for his belief that the mercury separates itself from the rest of the filling. From a chemical point of view we have reason to believe that there is too strong an affinity between the mercury and the alloy to admit of this taking place. In answer to Dr. Andrews's question, I believe there is more mercury in copper amalgam than there is in other alloys. A great fault with copper amalgam is in its manufacture. I became quite interested in copper amalgam through a paper which was read by Dr. Wegant some ten or twelve years ago, and in my study of the subject I found there was a trick in the manufacture in order to produce the desired result. It is the practice of the manufacturers to express the mercury from the copper amalgam by hydraulic pressure, and after doing this, when offering it for sale, if it was not plastic enough, they simply advised adding more mercury to it.

We know that by hammering an amalgam filling during its insertion it will express the mercury, and undoubtedly the pressure which we use in inserting the filling will cause the minute particles of mercury to penetrate the tubuli, as Dr. Andrews has stated.

I cannot understand what Dr. Briggs refers to when he speaks of the "high grade" alloys. According to Dr. Black's experiments, the best amalgam consists of silver, tin, and copper. Gold is a detriment to amalgam, and is simply put in to bring up the expense, and, being more expensive, many dentists infer that it must be better to use. It simply shows their ignorance of the necessary qualities of an amalgam.

I may say a little in the way of criticism of the essay. The writer has not given us any proof that the ill effect which he describes can be positively traced to mercury. We get the same effects from the poison of other metals; we get it from lead; we get it from copper. We know that mercury is often adulterated with lead, and in view of all this, in cases where there is an idiosyncrasy, the poisoning could result from copper and lead as well as from mercury, and we have not yet heard any evidence that such is not the case. Now, I have had a number of these symptoms that Dr. Taft has spoken of resulting from other causes to which I could trace them. I have seen these inflamed throats and mouths in cases where the patient was suffering from stomach trouble; seen them in mouths where there were no amalgam fillings whatever. I have one patient who presents these symptoms occasionally, and the use of trichloracetic acid cures it at once.

*Dr. Smith.*—My excuse for speaking again is because I wish to correct a statement of the last speaker that we did not want to have it go out from this Academy that we used amalgam on account of its cheapness. This Academy desires the facts to go forth, and I repeat that it is a very important fact, which we wish every one to know, that if the bill now pending before the Legislature, or soon to be brought before it, should pass, it would work a hardship to poor people, and we are willing to admit that in many cases we do use amalgam fillings on account of the expense to the patient. To cite a case: Suppose we have a patient whom we know to be very, very poor, and we have a large buccal cavity to fill for that patient. The patient cannot afford to pay for the time which it will require to put in a gold filling, and there are only two things to do,—fill the cavity entirely with cement, or entirely with amalgam. Now, there is no doubt that if the patient was to be deprived of the benefits of amalgam in that case, it would be a hardship, and we know that there are many, many such cases.

WILLIAM H. POTTER, D.M.D.,
*Editor American Academy of Dental Science.*

